# Decreased Levels of Serum IL-34 Associated with Cognitive Impairment in Vascular Dementia

**DOI:** 10.1155/2021/6793860

**Published:** 2021-05-21

**Authors:** Yang Wang, Wei Lu, Wenjing Ning, Yan Chen, Lingxing Li

**Affiliations:** ^1^Department of Neurology, Qingpu Branch of Zhongshan Hospital, Fudan University, Shanghai 201700, China; ^2^Department of Intensive Rehabilitation, Shandong Provincial Third Hospital, Cheeloo College of Medicine, Shandong University, Jinan, Shandong Province 250000, China; ^3^Department of Geriatric, Shandong Provincial Hospital, Cheeloo College of Medicine, Shandong University, Jinan, Shandong Province 250021, China; ^4^Department of Neurology, Jinan First People's Hospital, Shandong Traditional Chinese Medicine University, Jinan, Shandong Province 250013, China; ^5^Department of Geriatric Medicine, Shandong Provincial Third Hospital, Cheeloo College of Medicine, Shandong University, Jinan, Shandong Province 250000, China; ^6^Department of Cardiovascular Medicine, Taian City Central Hospital, Shandong First Medical University & Shandong Academy of Medical Sciences, Taian, Shandong Province. 271000, China

## Abstract

**Objective:**

Interleukin- (IL-) 34 is a new type of cytokine with neuroprotective effects discovered in recent years. However, the relationship between IL-34 and vascular dementia (VaD) has not yet been elucidated. The purpose of this study is to determine whether IL-34 is involved in cognitive impairment of VaD.

**Methods:**

From January 2017 to December 2020, 84 VaD patients and 60 healthy controls who attended Qingpu Branch of Zhongshan Hospital were prospectively included in the study. Once included in the study, demographic features of all research subjects are collected. They include age, gender, education, white blood cells (WBC), neutrophil, lymphocyte, systolic blood pressure (SBP), diastolic blood pressure (DBP), fasting blood glucose (FBG), triglycerides (TG), and total cholesterol (TC). Meanwhile, the Montreal Cognitive Assessment (MoCA) scale was used to assess the cognitive function of participants. The serum IL-34 level was determined by enzyme-linked immunosorbent assay (ELISA).

**Results:**

There was no significant difference between the demographic features of VaD patients and healthy controls (*p* > 0.05). However, the serum IL-34 levels of VaD patients and healthy controls are 27.6 ± 3.9 pg/ml and 41.8 ± 6.0 pg/ml, respectively, and there is a significant statistical difference between them (*p* < 0.001). The results of bivariate correlation analysis showed that serum IL-34 levels were significantly positively correlated with MoCA scores (*r* = 0.371, *p* = 0.023). Further regression analysis showed that IL-34 was still correlated with MoCA after adjusting for demographic features (*β* = 0.276, *p* = 0038).

**Conclusions:**

Serum IL-34 levels in VaD patients were significantly reduced, which may be an independent predictor of cognitive impairment in VaD patients.

## 1. Introduction

Vascular dementia (VaD) is a common type of dementia. The main cause of VaD is cerebrovascular disease, which leads to impaired cerebral blood flow and further damages neurons in the hippocampus or cortex [1, 2]. According to statistics from the World Health Organization (WHO), there are currently about 36 million people with dementia in the world. By 2050, this number will reach to 115 million, and VaD accounts for about 20% of them, second only to Alzheimer's disease (AD) [3]. It is reported that the prevalence of VaD in developed countries ranges from 1% to 4%, while the prevalence of VD in developing countries is about 8%, and the prevalence of VD in the elderly is rising rapidly [4]. The annual cost of VaD is as high as 200 billion US dollars, and with the extension of human life expectancy, VaD has gradually become one of the main health problems that need to be solved urgently in countries all over the world [5]. Our existing treatment may alleviate some of the symptoms of VaD in the short term, but it cannot fundamentally slow down the disease progression or reverse the disease process [6]. Therefore, it is particularly urgent to continue to explore the potential pathogenic mechanism and therapeutic targets of VaD.

Interleukin- (IL-) 34 was first discovered by Lin's team in 2008 and is considered to be a nonspecific ligand for colony stimulating factor- (CSF-) 1 receptor (CSF-1R) [7]. In addition to CSF-1R, IL-34 can also bind to protein-tyrosine phosphatase- (PTP-) *ζ* and syndecan-1. IL-34 is a homoglycan protein secreted mainly by brain neuron cells and skin keratinocytes, and it is highly conserved in vertebrates [8]. The human IL-34 gene is located on the 16q22.1 chromosome, while the mouse IL-34 gene is located on the 8E1 chromosome, which has high homology among different species [9]. More and more evidences show that IL-34 is involved in the etiology of various diseases, such as inflammation, infection, autoimmune diseases, and tumors [10].

Recent studies have shown that IL-34 has a certain neuroprotective effect in certain special diseases. However, it is not clear whether IL-34 is involved in the disease process of VaD. Therefore, the main purpose of our study is to explore the relationship between IL-34 and cognitive impairment in VaD patients, in order to provide potential targets and drugs for the treatment of VaD.

## 2. Methods

### 2.1. Study Participants

From January 2017 to December 2020, 84 VaD patients treated at Qingpu Branch of Zhongshan Hospital were included in the study. At the same time, 60 age- and gender-matched healthy controls were included. The diagnosis of VaD was completed by experienced physicians with reference to the National Institute of Nervous System Diseases and Stroke (NINDSAIREN) and the 11th edition of the International Classification of Diseases (ICD-11) [11, 12]. The exclusion criteria are as follows: combined with AD or Lewy body dementia; severe infectious diseases; taking anti-infective drugs; severe trauma; recent history of surgery; suffering from severe liver disease, kidney disease, or tumor; alcoholism; and severe mental illness. The patients or family members are written informed of the current research and agree to participate in the research. The study was approved by the ethics committee of our hospital and abides by the Declaration of Helsinki.

### 2.2. Demographic Features of the Participants

Of the 144 study participants, 84 were VaD patients, and the other 60 were healthy controls. The demographic features of all participants were carefully recorded by dedicated personnel, including age, gender, education, white blood cells (WBC), neutrophil, lymphocyte, systolic blood pressure (SBP), diastolic blood pressure (DBP), fasting blood glucose (FBG), triglycerides (TG), and total cholesterol (TC). Demographic feature information is obtained in the form of questionnaires.

### 2.3. MoCA Scale Evaluation

The Montreal Cognitive Assessment (MoCA) is used as a tool to evaluate cognitive function. Dr. Ziad Nasreddine of Canada invented the MoCA scale in 1996, which was later translated into multiple languages and widely used because of its good accuracy. MoCA evaluated the following cognitive areas: short-term memory, visuospatial abilities, executive functions, attention, language, abstract reasoning, and orientation. The MoCA score ranges from 0 to 30, and a score of 26 or more is considered normal [13, 14].

### 2.4. Serum ELISA Test

Enzyme-linked immunosorbent assay (ELISA) is a popular immunoassay method that can quickly and easily analyze the same indicator in a large number of samples. In this experiment, we used an ELISA kit (MyBioSource, San Diego, CA, USA) to detect the concentration of IL-34 in the serum. Fasting venous blood of all participants was collected within 24 hours of enrollment. The collected whole blood was placed in the refrigerator overnight at 4°C and then centrifuged at 3000 rpm for 10 minutes, and the supernatant was immediately taken to be tested and stored in the refrigerator at -80°C. The specific experimental steps refer to previous reports and product manuals [15].

### 2.5. Statistical Analysis

In this study, categorical variables are represented by numbers, and continuous variables are represented by mean ± standard deviation. The comparison between the two groups used *t*-test or chi-square test. Spearman's rank was used to assess the bivariate correlation, and multivariate regression analysis was used to clarify the causal relationship between the variables. All data were analyzed using the SPSS 22.0 software (SPSS Inc., IL, USA), and a two-tailed *p* value < 0.05 was considered statistically significant.

## 3. Results

### 3.1. Demographic Features of the Participants

**Figure 1:**
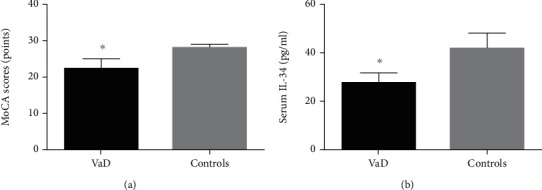
The MoCA scores and serum IL-34 levels of VaD patients and healthy controls. (a) MoCA scores; (b) serum IL-34 levels. Compared with the controls, ^∗^*p* < 0.05.

Of the 144 study participants, 84 were VaD patients, and the other 60 were healthy controls. The demographic features of the participants are shown in Table 1. There was no significant difference between the demographic features (age, gender, education, WBC, neutrophil, lymphocyte, SBP, DBP, FBG, TG, and TC) of VaD patients and healthy controls (*p* > 0.05). However, the serum IL-34 levels of VaD patients and healthy controls are 27.6 ± 3.9 pg/ml and 41.8 ± 6.0 pg/ml, respectively, and there is a significant statistical difference between them (*p* < 0.001). The comparison of the MoCA score and serum IL-34 levels of VaD patients and healthy controls is shown in Figure 1.

### 3.2. Bivariate Correlation Analysis

In order to clarify the potential factors affecting the cognitive function of VaD patients, we conducted a bivariate correlation analysis of the MoCA score and the variables of the demographic features. The results of the bivariate correlation analysis are summarized in Table 2. From the results in Table 2, it can be seen that the MoCA score of VaD patients is significantly positively correlated with serum IL-34 levels (*r* = 0.371, *p* = 0.023). However, there is no significant correlation between MoCA score and age, gender, education, WBC, neutrophil, lymphocyte, SBP, DBP, FBG, TG, and TC (*p* > 0.05).

### 3.3. Multivariate Regression Analysis

In order to understand the relationship between IL-34 levels and the cognitive function of VaD patients, we conducted a multivariate logistic regression analysis between the MoCA score and demographic features including IL-34. The results of the multivariate logistic regression analysis are summarized in Table 3. After adjusting for various demographic features, the relationship between IL-34 and the cognitive impairment of VaD patients is still significant, with an OR of 2.171 (*β* = 0.276, *p* = 0038). These results indicate that IL-34 levels are positively correlated with VaD MoCA scores. In other words, VaD patients with low serum IL-34 levels may have more severe cognitive impairment.

## 4. Discussion

This study mainly explored the correlation between the cognitive function of VaD patients and the level of serum IL-34. The preliminary results showed that the serum IL-34 level and MoCA score of VaD patients were significantly lower than those of healthy controls. The results of correlation analysis showed that the MoCA score of VaD patients was significantly positively correlated with serum IL-34 levels and was not related to age, gender, education, WBC, neutrophil, lymphocyte, SBP, DBP, FBG, TG, and TC and other demographic features. Regression analysis, after correcting for interference factors, further confirmed the correlation between serum IL-34 and MoCA in VaD patients. As far as we know, this is the first research report that uses serum IL-34 as an index to predict cognitive impairment in VaD patients.

Cytokines are a group of secreted proteins, and its members include interleukins, lymphokines, chemokines, tumor necrosis factor, and interferon. Cytokines produced by a variety of cells can mediate cell-cell signal transduction through autocrine and paracrine and play a key role in the communication between different cells, tissues, and organs. The combination of cytokines and specific receptors not only controls the growth, proliferation, and migration of main cells at the cellular level but also participates in the body's inflammation, tumor, immunity, and angiogenesis [16]. Therefore, research on cytokines as diagnostic markers and therapeutic targets has been in the ascendant for a long time, and some progress has been made in predicting the severity and prognosis of the disease. IL-34 was identified as the second ligand of CSF1R in 2008. The full length of human IL-34 contains 242 amino acids. The first 182 amino acids are essential for the stability and proper folding of IL-34, and the last 50 residues are a large number of disordered and Pro-Ser-Thr-rich amino acids [17]. It has a similar active region to CSF-1, but their sequence does not have homology. IL-34 is a hematopoietic cytokine, which is widely distributed in the neurons, skin, heart, lung, liver, kidney, testes, and lymph nodes. It is a key regulator of the survival, proliferation, and differentiation of myeloid cells [18].

Recent studies have found that IL-34 is involved in the process of many diseases. Scholars from Dalian Medical University found that IL-34 increases the expression of IL-6 and then upregulates Th17 to participate in the course of rheumatoid arthritis [19]. Researchers from Chongqing Medical University found that serum IL-34 is elevated in patients with systemic lupus erythematosus (SLE), which may be used as a diagnostic marker for SLE [20]. In addition, Polish studies have shown that IL-34 can be used as a potential inflammatory biomarker for predicting the risk of diabetes complications [21]. Interestingly, the scientific research team of Shanghai Jiao tong University found that increased levels of IL-34 in the acute phase are associated with increased risk of heart failure and poor prognosis after myocardial infarction [22]. IL-34 is also believed to be related to psoriasis, psoriatic arthritis, obesity, liver disease, kidney disease, and inflammatory bowel disease [23–28].

The role of IL-34 in neurological diseases has gradually attracted attention in recent years. IL-34 can act on a variety of cells such as neurons, microglia, and endothelial cells to maintain the homeostasis of the central nervous system [29, 30]. A professor at Stanford University found that IL-34 provides a powerful neuroprotective and survival signal for neurons in brain injury and neurodegeneration [31]. Recently, Iranian scientists have found that serum IL-34 levels in patients with acute and chronic inflammatory demyelinating polyneuropathies (AIDP and CIDP) are elevated, suggesting that IL-34 may participate in the pathogenesis of autoimmune diseases [32]. In addition, West Nile virus infection can cause cognitive impairment, and IL-34 knockout mice can reduce the synaptic loss caused by this virus infection, suggesting that IL-34 may participate in the course of cognitive impairment [33]. Canadian researchers initially revealed the mechanism by which IL-34 is involved in the pathogenesis of AD [34], but its relationship with VaD has not been reported yet.

Our research has some limitations. First of all, we are a single-center small sample study; secondly, our VaD patients may have mixed types of dementia; and finally, we have not done interventional mechanism research. However, we have studied the relationship between serum IL-34 and VaD for the first time, which is the advantage of our research.

## 5. Conclusions

In summary, we found that serum IL-34 levels in VaD patients were significantly reduced. We report for the first time the role of serum IL-34 in VaD. IL-34 may be used in clinical services as a diagnosis and treatment target of VaD.

## Figures and Tables

**Table 1:** Demographic features of the participants.

	VaD (*N* = 84)	Controls (*N* = 60)	*p* value
Age (year, mean ± SD)	70.3 ± 8.9	70.5 ± 9.2	0.896
Gender (male/female)	50/34	38/22	0.644
Education (year, mean ± SD)	8.4 ± 1.5	8.6 ± 1.8	0.469
WBC (×10^9^/L, mean ± SD)	7.6 ± 1.3	7.4 ± 1.6	0.410
Neutrophil (×10^9^/L, mean ± SD)	5.2 ± 1.4	5.0 ± 1.1	0.358
Lymphocyte (×10^9^/L, mean ± SD)	1.8 ± 0.6	1.9 ± 0.7	0.359
SBP (mmHg, mean ± SD)	140.6 ± 12.2	139.8 ± 12.9	0.705
DBP (mmHg, mean ± SD)	87.7 ± 8.8	87.1 ± 8.5	0.683
FBG (mmol/L, mean ± SD)	7.3 ± 0.6	7.1 ± 0.8	0.089
TG (mmol/L, mean ± SD)	1.6 ± 0.2	1.6 ± 0.1	1.000
TC (mmol/L, mean ± SD)	4.6 ± 0.5	4.5 ± 0.4	0.202
MoCA (points, mean ± SD)	22.5 ± 2.6	28.1 ± 1.2	<0.001
IL-34 (pg/ml, mean ± SD)	27.6 ± 3.9	41.8 ± 6.0	<0.001

Abbreviations: VaD, vascular dementia; WBC, white blood cells; SBP, systolic blood pressure; DBP, diastolic blood pressure; FBG, fasting blood glucose; TG, triglycerides; TC, total cholesterol; MoCA, Montreal Cognitive Assessment; IL-34, interleukin-34.

**Table 2:** Correlation between demographic features and MoCA in VaD.

	*r*	*p* value
Age (year, mean ± SD)	-0.287	0.134
Gender (male/female)	0.329	0.399
Education (year, mean ± SD)	-0.173	0.097
WBC (×10^9^/L, mean ± SD)	0.418	0.283
Neutrophil (×10^9^/L, mean ± SD)	0.556	0.431
Lymphocyte (×10^9^/L, mean ± SD)	0.314	0.262
SBP (mmHg, mean ± SD)	-0.205	0.305
DBP (mmHg, mean ± SD)	-0.231	0.338
FBG (mmol/L, mean ± SD)	-0.270	0.196
TC (mmol/L, mean ± SD)	-0.402	0.583
TG (mmol/L, mean ± SD)	-0.357	0.677
IL-34 (ng/ml, mean ± SD)	0.371	0.023

Abbreviations: MoCA, Montreal Cognitive Assessment; VaD, vascular dementia; WBC, white blood cells; SBP, systolic blood pressure; DBP, diastolic blood pressure; FBG, fasting blood glucose; TG, triglycerides; TC, total cholesterol; IL-34, interleukin-34.

**Table 3:** Multivariable analyses of demographic features and MoCA in VaD.

	Regression coefficient	*p* value
Age (year, mean ± SD)	0.213	0.569
Gender (male/female)	0.217	0.117
Education (year, mean ± SD)	0.139	0.209
WBC (×10^9^/L, mean ± SD)	0.296	0.318
Neutrophil (×10^9^/L, mean ± SD)	0.245	0.167
Lymphocyte (×10^9^/L, mean ± SD)	0.197	0.388
SBP (mmHg, mean ± SD)	0.115	0.271
DBP (mmHg, mean ± SD)	0.168	0.226
FBG (mmol/L, mean ± SD)	0.103	0.309
TC (mmol/L, mean ± SD)	0.186	0.275
TG (mmol/L, mean ± SD)	0.153	0.224
IL-34 (ng/ml, mean ± SD)	0.276	0.038

Abbreviations: MoCA, Montreal Cognitive Assessment; VaD, vascular dementia; WBC, white blood cells; SBP, systolic blood pressure; DBP, diastolic blood pressure; FBG, fasting blood glucose; TG, triglycerides; TC, total cholesterol; IL-34, interleukin-34.

## Data Availability

The data used to support the findings of this study are available from the corresponding authors upon request.
